# Common Medications Which Should Be Stopped Prior to Platelet-Rich Plasma Injection

**DOI:** 10.3390/biomedicines10092134

**Published:** 2022-08-31

**Authors:** Ashim Gupta, Madhan Jeyaraman, Nicola Maffulli

**Affiliations:** 1 Future Biologics, Lawrenceville, GA 30043, USA; 2 BioIntegrate, Lawrenceville, GA 30043, USA; 3 South Texas Orthopaedic Research Institute, STORI Inc., Laredo, TX 78045, USA; 4 Indian Stem Cell Study Group (ISCSG) Association, Lucknow 110048, India; 5 Department of Orthopaedics, Faculty of Medicine, Sri Lalithambigai Medical College and Hospital, Dr. MGR Educational and Research Institute University, Chennai 600095, India; 6 Department of Musculoskeletal Disorders, School of Medicine and Surgery, University of Salerno, 84084 Fisciano, Italy; 7 San Giovanni di Dio e Ruggi D’Aragona Hospital “Clinica Ortopedica” Department, Hospital of Salerno, 84124 Salerno, Italy; 8 Barts and the London School of Medicine and Dentistry, Centre for Sports and Exercise Medicine, Queen Mary University of London, London E1 4DG, UK; 9 School of Pharmacy and Bioengineering, Keele University School of Medicine, Stoke on Trent ST5 5BG, UK

Osteoarthritis (OA) is an extremely prevalent joint condition in the United States, affecting over 30 million people [1]. Its pathophysiology is linked with inflammation of the synovial tissue and degeneration of articular cartilage, resulting in pain and decreased function [2,3,4]. OA normally affects larger weight-bearing joints, with the number of people suffering from knee OA anticipated to reach 67 million by 2030 [1]. Normally, OA is managed with activity modification, physical therapy, pharmacological agents (such as, NSAIDs, corticosteroids, viscosupplementation, opioids, etc.), and surgery after conservative management modalities have failed [5]. These treatment options have limitations, continually trying to reduce pain as opposed to aiming on the underlying pathology [5,6]. 

Over the last decade, a number of molecular targets, such as interleukin-1 (IL-1), transforming growth factor-β (TGF-β), matrix metalloproteinases (MMPs), etc., have been identified as being involved in the etiopathogenesis of OA [7,8,9], yet many treatments may well have a negative risk-to-benefit ratio [10,11]. Thus, other safe and effective treatment options are required to address this unmet medical need. 

Recently, there has been a notable growth in the use of biologics, including platelet-rich plasma (PRP), for regenerative medicine applications, particularly in musculoskeletal medicine [12]. PRP is an autologous blood-derived product containing patients’ own concentrated platelets in a small volume of plasma utilized for treatment of several conditions [13,14]. PRP exerts its effect on the adjacent tissue following release of a variety of growth factors and cytokines from the platelet concentrate [15]. 

Two factors—platelet count and platelet aggregation—have been indicated to affect the efficacy of PRP. No consensus exists for a standardized concentration of platelets in the PRP, and studies have demonstrated that too low or too high of a platelet count can hinder the efficacy of PRP [16]. Notably, a recent study reported that the platelet count was positively correlated with the concentration of growth factors [17]. This demonstrated that the platelet count plays a vital role in determining the capability of PRP to exert its downstream effects. Similarly, platelet aggregation is essential for platelets to secrete their growth factors via a process of intracellular protein phosphorylation [15,18]. 

Despite much research on preparation and activation methods of PRP [19,20], there is insufficient literature on the efficacy of PRP injections according to patient-related variables. One study identified this gap and attempted to recognize patient-specific variables that could influence the PRP effectiveness [21]. In particular, this study identified medications, mental and physical stress levels, blood pressure, smoking status, and alcohol consumption as patient-specific variables that should be considered given their ability to affect the analgesic efficacy of PRP [21]. Nevertheless, there are limited number of studies on common medications consumed by patients and their effects on PRP. Additionally, there is a lack of detailed guidelines pertaining to which medications should be stopped prior to PRP injection. Here, the author focused on a recently published review [22] that evaluated some of the most commonly prescribed medications in the US and their effects on PRP, in order to establish guidelines for medications that need to be stopped prior to a PRP injection. 

Paracetamol induced a profound diminished aggregation of platelets when compared with placebo in a dose-dependent manner [23,24]. Patients treated with non-selective NSAIDs, such as ibuprofen or indomethacin, demonstrated no change in platelet count but decreased platelet aggregation was noted. Patients who took diclofenac after recent orthopaedic procedures demonstrated substantial reduction of aggregation of platelets [25]. Decreased platelet aggregation was observed with a single dose of intravenous diclofenac with decreasing levels of TxB2 to 1.6% of baseline [26]. After a recent orthopaedic procedure, PRP samples drawn from patients who took dexibuprofen or diclofenac showed a significant decrease in aggregation of platelets when compared with placebo [25]. Decreased platelet aggregation and serum TxB2 concentration were observed in healthy volunteers treated with 500 mg naproxen twice daily for 10 days [27]. A complete reversal of platelet aggregation was demonstrated within 24 h with single dose of sulindac; however, with continuous administration for 8 days, platelet aggregation remained inhibited [28]. Supratherapeutic dose of valdecoxib (40 mg BD) and therapeutic dose of naproxen (500 mg BD) and diclofenac (75 mg BD) do not interfere in the function of platelets in healthy volunteers [29]. Meloxicam decreased TxB2 production in a dose-dependent manner when compared with placebo [30]. In patients who take daily low dose aspirin, Jayaram et al. demonstrated reduced growth factors levels in freshly prepared human leucocyte rich-PRP when activated with arachidonic acid [31].

Anitua et al. confirmed the reduced angiogenic potential and the capacity to induce hyaluronic acid and fibronectin of PRP in patients taking anticoagulants. The biological potential of platelet-rich growth factors is maintained in patients taking acetylsalicylic acid, acenocoumarol, glucosamine sulfate and chondroitin sulfate [32]. Cellular proliferation was not affected, whereas cellular migration was enhanced by acetylsalicylic acid, acenocoumarol, glucosamine sulfate and chondroitin sulfate. No effect on extracellular matrix proteins secreted by gingival fibroblasts was noted [33]. Ketorolac and PRP increases chondrocyte and tenocyte viability than methylprednisolone [34]. COX-2 inhibitors do not inhibit platelet activation or growth factor release from PRP [35]. Leucocyte rich PRP samples from patients using naproxen demonstrate a diminished PDGF and IL-6 levels without affecting TNF-α, IL-1β, IL-8, VEGF, and FGF-2. All factors were normalized after a 1-week washout period [36]. There was no reduction in the release of anabolic growth factors when patients used acetylsalicylic acid or acetylsalicylic acid with clopidogrel [37]. NSAIDs such diclofenac and meloxicam did not alter the release of VEGF and PDGF-AB levels of PRP [38]. There was no difference in thrombin production and platelet activation in response to TRAP-6, but there was significantly decreased ADP-induced platelet activation [39].

When administering PRP as a therapeutic agent for musculoskeletal disorders, the treating physician/surgeon must be aware of the quality of PRP being delivered at the target site, the factors responsible for procuring quality PRP, and the medications interfering with the homeostasis of PRP. 

## Data Availability

Not applicable.
